# Reductive prodrug and AIE copolymer nanoparticle for monitoring and chemotherapy

**DOI:** 10.1186/s12885-024-12135-7

**Published:** 2024-03-26

**Authors:** Zigui Wang, Guilin Li, Qiaohui Zhao, Guangyu Fu, Zengli Yang, Guojun Zhang

**Affiliations:** 1 Department of Clinical Diagnosis, Laboratory of Beijing Tiantan Hospital, Capital Medical University, Beijing, China; 2 NMPA Key Laboratory for Quality Control of In Vitro Diagnostics, Beijing, China; 3 Beijing Engineering Research Center of Immunological Reagents Clinical Research, Beijing, China; 4 Zhengzhou Immunobiotech Co, Ltd, Zhengzhou, 450016 P.R. China; 5 Autobio Diagnostics Co, LtdHenan, 450016 China

**Keywords:** AIE, Chemotherapy, Platinum, Drug delivery, Real-time tracing

## Abstract

Polymeric micelle systems for drug delivery, monitor and chemotherapy have gained significant attention, and reductive polymeric micelle systems have become particularly attractive due to their controlled release behavior without additional assistance. However, there are challenges in accurately controlling drug and probe release from the nanoparticles and determining the loading content of drug and probe. To address these issues, we have developed a reduction-responsive Pt(IV) prodrug-based polymeric delivery system that can be dynamically monitored using aggregation-induced emission luminogens (AIE) based bioprobes. These polymeric micelle can self-assemble into nanoparticles and release both bio-active Pt(II) drug and bio-probe upon reduction activation. TPE molecules released in the inner endo/lysosomal microenvironment aggregate and fluoresce upon irradiation, thus allowing real-time tracking of drug biodistribution without additional contrast agents. Advantages of this system include position-specific chemical bond cleavage, control of platinum content, and monitoring of drug reduction and biodistribution.

**Supplementary Information:**

The online version contains supplementary material available at 10.1186/s12885-024-12135-7.

## Introduction

Recently, there has been a surge in interest in advanced polymeric micelle delivery systems for controlled and targeting chemotherapy and simultaneous drug tracking [1, 2]. Various stimuli, such as pH, redox, oxidative stress, temperature, and light, have been utilized to control drug release in these systems [3–7]. Among these, reductive polymeric micelle systems have attracted great attention due to their low toxicity and ability to achieve controlled release without ex vivo stimuli or assistance. Current design for formation of prodrug nanoparticles are mainly about drugs encapsulated into nanoparticles with stimuli molecules on the key chemical bond of polymer or conjugation of the drug to the polymer through a linker to form prodrug nanoparticles are among the most common strategies [8, 9]. However, these traditional polymeric micelles may exhibit variable drug release behaviors, posing challenges for precise release control within the body (in vivo). Additionally, the loading content of drugs remains uncertain with common encapsulation methods, which complicated drug release in body or targeted cancer regions [10]. Furthermore, real-time monitoring of drug distribution in the body is currently not feasible. The use of fluorescent agents or other contrast agents for diagnostic imaging in therapeutics increases the complexity and cost of synthesis or manufacturing and can lead to unforeseen adverse side effects [11–13]. These challenges significantly limit the widespread application of polymeric micelle systems and new design or synthetic strategies are highly needed for more precisely control and real-time monitoring [14].

Another challenge in real-time tracking and therapy systems is the inability to precisely monitor the time it takes for drug concentrations to accumulate at tumor sites and other organs, such as the heart, liver, spleen, lung, and kidney [15, 16]. With this regard, we hypothesize that integrating fluorescent molecules into reduction-responsive polymeric delivery systems could enable dynamic monitoring of drug biodistribution. Among all the current imaging diagnostic techniques, there are mainly fluorescence imaging, positron emission tomography (PET)/CT and magnetic resonance imaging (MRI) [17–20]. Due to CT’s high resolution and deep tissue penetration, it is the most commonly used diagnostic technique in clinical practice. However, used small molecular CT contrast agents like iohexol and iodixanol were difficult to conjugated it to polymer. Besides, these have drawbacks such as rapid blood clearance, high viscosity, and high osmolality, which limited its application [21, 22]. Some fluorescence molecules can be utilized to construct fluorescent nanoprobe with low toxicity and minimal side effects but traditional dyes often suffer from aggregation-caused quenching (ACQ) when assembled into aggregate [23–26]. On the contrary, bioprobes based on aggregation-induced emission (AIE) exhibit attractive features in monitoring cell molecules with huge potential [22]. Recently, AIE has been polymerized into polymer backbone to develop AIE polymeric nanoparticles for drug delivery [7]. Nevertheless, the fluorescence of AIE polymeric nanoparticles is consistently active everywhere without specificity due to the intramolecular motion mechanism of AIE. To achieve in situ activation of AIE polymeric nanoparticles at target sites, intricate molecular design and modification are required for the system.

**Scheme 1:**
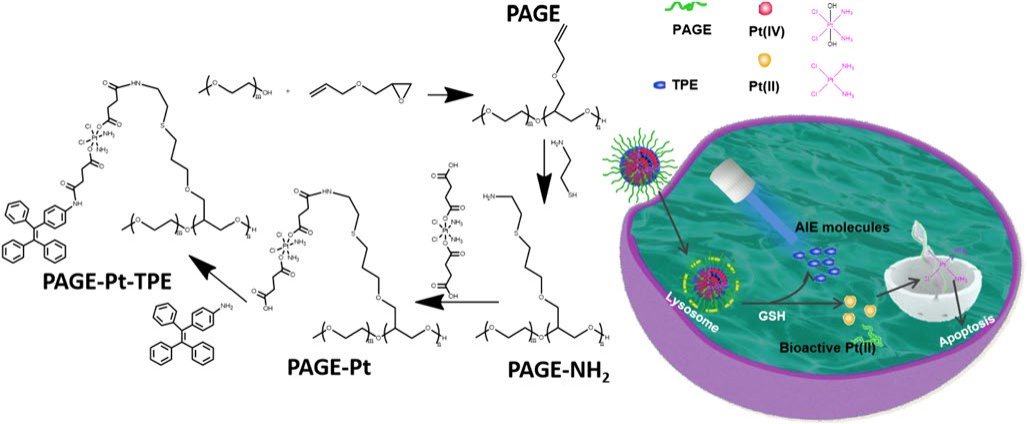
Schematic diagram of reductive prodrug and AIE copolymer micelles for monitoring and chemotherapy

Recently, we have synthesised a series of reductive Pt(IV) prodrug-based polymeric delivery systems for low side chemotherapy [2, 27]. However, dynamically monitoring the spatiotemporal distribution of drugs at tumor sites and other organs remains challenging. As a cytotoxic platinum( II) (Pt( II)) prodrug, a hydrophobic platinum( IV) (Pt( IV)) complex has many advantages, such as higher stability and lower cytotoxicity, which could undergo reduction to bio-active Pt( II) inside cancer cells [28–31]. Thus, a Pt( IV) complex can be conjugated to polymeric key joint and be released in a controlled fashion at tumor site, serving as both a drug and reduction-responsive moiety [32]. To combine the advantages of aggregation-induced emission luminogens based bioprobes and Pt(IV) complexes, we constructed polymeric micelles by conjugating hydrophobic Pt(IV) inside the polymeric chain and the monitor molecules TPE conjugated on the Pt(IV) molecules, which can self-assemble into nanoparticles (PAGE-Pt-TPE) in water solution. After cancer cells swallow PAG-PT-TPE, the chemical bond between the polymer and Pt(IV) will be broken by reduction of GHS, and the Pt(IV) prodrug in the core of PAG-PT-TPE will be activated to release the active Pt(II) drug, including DNA damage. Subsequently, TPE released under the acidic endo/lysosome microenvironment would aggregate and fluoresce upon irradiation, thereby enabling real-time tracing (Scheme 1). The PAGE-Pt-TPE system possesses several advantages: (1) Activation of tumor site-specific chemical bond cleavage by reducing agents triggers release of Pt(II) drugs and biological probes without the introduction of additional ACQ. (2) Prodrug Pt(IV) conjugated in polymer chain side enable precise control the content of Pt. (3) PAGE-Pt-TPE is expected to be able to monitor the cleavage of polymeric micelle. The reduction of prodrug Pt(IV) to active Pt(II) was monitored to dynamically monitor the biodistribution of the drug without additional contrast agents. Additionally, the fluorescence intensity can also reflect the sensitivity of tumor cells to drugs.

## Experiment

### Materials and Measurements

Cisplatin was bought from Shandong Boyuan Chemical Company, China.1-(4-Aminophenyl)-1,2,2-triphenylethene, N-hydroxysuccinimide (NHS), pyridine, 1-ethyl-3-(3-dimethylaminopropyl) carbodiimide hydrochloride (EDC), methoxy polyethylene glycol (Mn = 8000 Da, mPEG_8k_), 3-(4,5-dimethylthiazol-2-yl)-2,5-diphenyltetrazolium bromide (MTT), Allyl glycidyl ether (AGE), 2,2'-Azobis(2-methylpropionitrile) (AIBN), succinic anhydride 1-(4-Aminophenyl)-1,2,2-triphenylethene (TPE) and 2-Aminoethanethiol hydrochloride were purchased from Aladdin Chemistry Co. Ltd. (Shanghai, China). All other reagents were commercially available and used as received.

### Measurements

Proton nuclear magnetic resonance (^1^HNMR) spectra were measured on a Bruker AVANCE DRX 400 spectrometer using deuterated dimethyl sulfoxide (DMSO d_6_) and deuterium oxide (D_2_O). The molecular weight and polydispersity index of the synthetic polymer were detected using a Tetrahydrofuran 515 gel permeation chromatograph (GPC). Diameter and polydispersity index were measured on Malvern ZETASIZER LAB. Transmission electron microscope (TEM) images were collected using a JEOL JEM-1011 transmission electron microscope with an acceleration voltage of 100 kV. An inductively coupled plasma mass spectrometer (ICP-MS, Xseries II, Thermo Fisher Scientific, USA) was used for the analysis content of Pt. Electrospray ionization mass spectrometry (ESI–MS) measurement was conducted on a Waters Quattro Premier XE system. The molecular weight and polydispersity index of the synthetic polymer were determined using a Tetrahydrofuran 515 gel permeation chromatograph (GPC). The molecular weight of PAGE-Pt-TPE was analyzed by using Autof ms800 (Autobio Diagnostics, Zhengzhou, China) systems.

### Synthesis of Pt(IV) prodrug

Briefly, cisplatin (1 g) was suspended in H_2_O_2_ (20 mL, 30%) stirred for 24 h at room temperature. Finally, Pt(IV) was obtained by filtration and washing with water and ethanol.

### Synthesis of Pt(IV)-COOH

The Pt(IV) prodrug (1 g, 2.9 mmol) and succinic anhydride (1.168 g, 11.6 mmol) were dissolved in N,N-Dimethylformamide (DMF, 20 ml). After reaction at 60 ℃ for 24 h, solution was poured into diethyl ether and filtering it for three time.

### Synthesis of PAGE

Block copolymer PAGE was synthesized according to the literature with a slight change as shown in Scheme 1. Briefly, dried mPEG_8k_(2.0 mmol), 305 mg cesium hydroxide monohydrate (1.8 mmol, 0.9 equiv), and 40 mL dried toluene were added to a dry reaction bulb under argon/nitrogen atmosphere. In order to acquire partially deprotonated macroinitiator, it is necessary to evacuate at 90 ℃ for 3 h. After that, additional 100 mL of toluene was added and 6.84 g AGE (60.0 mmol, 15 equiv) was added. The polymerization reaction needs to be maintained for 48 h at 45 ℃ for 48 h under an inert atmosphere. To stop the polymerization was stopped, a trace of acetic acid–ethanol (50/50) solution was added.The reaction solution was poured into a mount of cold diethyl ether for 3 times and the polymers were collected and dried in vacuum for 8 h (85%).

### Synthesis of PAGE-NH_2_

PAGE (5 g), 2-Aminoethanethiol hydrochloride (10 g, 62.5 mmol) and dried 20 mL of DMF were add to dry reaction bulb under inert atmosphere. After dissolution and addition of AIBN, the reaction maintained for 24 h at 65 ℃ under an inert atmosphere. DMF was removed at 50 ℃ under vacuum. Residue was redissovled in CH_2_Cl_2_ and precipitated in cold diethyl ether. After filtered and dried, mPEG-NH_2_ was acquired.

### Synthesis of PAGE-Pt

In general, large excess of Pt(IV)-COOH prodrug (245 mg, 0.46 mmol), EDC (265 mg, 1.38 mmol), and NHS (159 mg, 1.38 mmol) were added into reaction bulb and 8 mL of DMF was injected. The solution was activated for 4 h in ice bath. After that, poured PEGA-NH_2_ (184 mg, 0.023 mmol) into reaction solution and maintained the reaction at 25 ℃ for 3 days. Finally, the solution was poured into Spectra/Por Regenerated Cellulose membrane (MWCO = 3500), which was dialyzed with purified water for 2 days. After freeze-dried, PAGE-Pt was obtained.

### Synthesis of PAGE-Pt-TPE

In general, PAGE-Pt polymeric prodrug (100 mg, 0.01 mmol), EDC (46 mg, 0.24 mmol), and NHS (28 mg, 0.24 mmol) were added into reaction bulb and 8 mL of DMF was injected. The solution was activated for 4 h in ice bath. After that, poured TPE (69.4 mg, 0.2 mmol) into reaction solution and maintained the reaction at 25 ℃ for 3 days. Finally, the solution was poured into Spectra/Por Regenerated Cellulose membrane (MWCO = 3500), which was dialyzed with purified water for 2 days. After freeze-dried, PAGE-Pt-TPE was obtained.

### Reductive drug release of PGAE-Pt-TPE

To simulate the release process of PAGE-Pt-TPE in vivo*.* PAGE-Pt-TPE (2 mg) was dissolved in different phosphate buffer, inluding 1 mL of PBS (0.01 M, pH 7.4), PBS (0.01 M, pH 7.4, 1 mg/ml SA), PBS (0.01 M, pH 5.0) or PBS (0.01 M, pH 5.0, 1 mg/ml SA). The solution was poured into a dialysis bag (MWCO = 500) and 19 mL of the corresponding PBS solutions was added. Drug release was conducted at 37 ℃ in an air bath shaker.

### Cell culture

Human cervical carcinoma HeLa cells were cultured with Dulbecco’s modified Eagle’s medium containing 10% fetal bovine serum (FBS). The U14 cells were cultured in the ascites of mice. These and murine tumor cells U14 were acquired from the Institute of Biochemistry and Cell Biology, Chinese Academy of Sciences, Shanghai, China.

### Cellular uptake of TPE

For TPE release in cells, The germfree coverslips were added into 6-well plates. HeLa cells were cultured on it (2 × 10 ^5^ cells per well) for 24 h. PAGE-Pt-TPE was dissolved in DMEM (Pt: 54 µM) and the initial medium was substituted with PAGE-Pt-TPE solution for 2 h and 4 h, respectively. After cultured for corresponding time, HeLa cells were washed with cold PBS. For CLSM observation, the HeLa cells on the coverslips were fixed using formaldehyde(4%) for 15 min 25 ℃. We observed fluorescence images using a CLSM.

### Cell viability

Cells were seeded in 96-well plates (5 × 10^3^ cells per well). After incubated in DMEM for 24 h, the medium was added with cisplatin, TPE, Pt(IV), PAGE-Pt and PAGE-Pt-TPE from 3.375 to 216 µM (final Pt concentration from or equal molar amount Pt). After incubating for 48 h, MTT was added, and 96-well plates was at incubator for another 4 h. Afer removal of MTT solution, 150 mL of DMSO was added to each well to dissolve the formazan crystals formed in the cells. After mixed for 5 min, the absorbance was acquired at 490 nm using a microplate reader.

### Animals used

Kunming (KM) mice were bought from Jilin University. The experimental mice bearing U14 xenograft tumor model were acquired by subcutaneously injecting U14 cells (1 × 10^6^, 0.1 mL PBS) into right legs of mice. All the in vivo study protocols were approved by the local institution review board and performed according to the Ethics Committee of the Beijing Tiantan Hospital of Capital Medical University, China. After volume of subcutaneous U14 tumor model reach to 500 mm^3^, the animals were anaesthetized with pentobarbital sodium and the tumor was collected from sacrificed mice. It was cut into pieces and treated with homogenizer and pressure cell disruptor. After that, supernatant was collected by centrifugation.

## Results and discussion

**Fig. 1:**
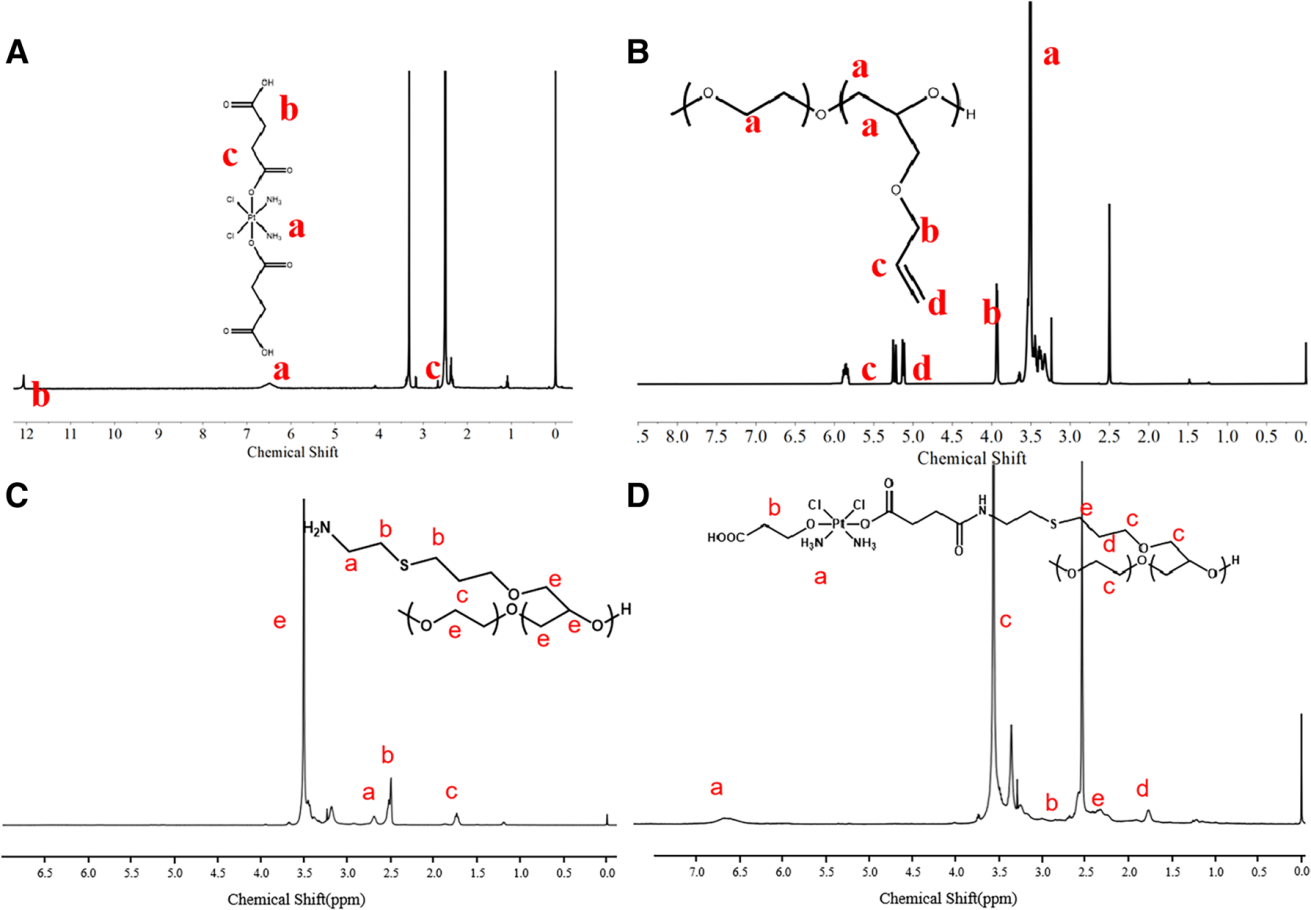
The characteristics of Pt(IV), PAGE, PAGE-NH_2_ and PAGE-Pt**.** ^1^H NMR spectrum of **A** Pt(IV), **B** PAGE, **C** PAGE-NH2 and **D** PAGE-Pt in DMSO

Pt(II) drugs have been extensively utilized in the treatment of various cancers over the past decades, yet their application has been hindered by severe side effects. Pt(IV) prodrugs, particularly those with reductive properties, have shown promise as a preferable option to mitigate toxic side effects. In this study, a reductive Pt(IV) prodrug was integrated with TPE as a fluorescence molecule, initially coupled with the Pt(IV) prodrug to produce amphiphilic PAGE-Pt-TPE.obtain amphiphilic PAGE-Pt-TPE. The successful synthesis of PAGE, PAGE-NH_2,_ PAGE-Pt was confirmed by ^1^H NMR and GPC was also conducted to characterize the molecular weight of PAGE-Pt (Mn = 14,500 Da, PDI = 1.13 Fig. S3). The peak at δ = 3.51 ppm could be assigned to –NH_3_ of Pt(IV) in DMSO d_6_, which indicates the successful synthesis of Pt(IV) (Fig. 1A). As can be seen in Fig. 1B, PAGE displayed the ^1^H NMR signals of mPEG (3.65 ppm), AGE (c at ~ 5.3 ppm, d at ~ 5.9 ppm, and b at ~ 4.0 ppm). After reaction with 2-aminoethanethiol hydrochloride, the ^1^H NMR all the signals of AGE (c at ~ 5.3 ppm, d at ~ 5.9 ppm, and b at ~ 4.0 ppm) disappeared in DMSO d_6_ (Fig. 1C, which indicates that PAGE-NH_2_ was prepared. To synthesis PAGE-Pt, PEGA-NH_2_ and Pt(IV) were stirred at room temperature for 3 days. As shown in Fig. 1D, PAGE-Pt displayed the ^1^H NMR signals of Pt(IV) (-NH_3_, 6.5 ppm).

**Fig. 2:**
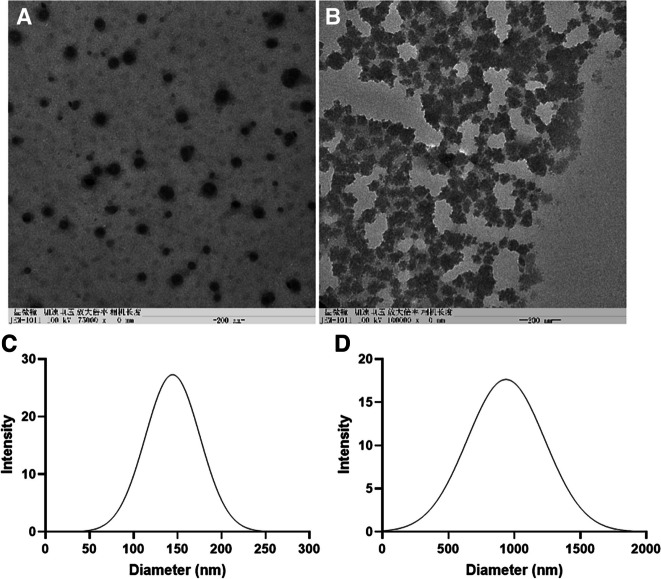
Characterization of reduction-sensitive self-assembled PAGE-Pt-TPE. TEM images of PAGE-Pt-TPE) before and **B** after treated with SA (5 × 10^–3^ M) for 24 h. Size distribution of PAGE-Pt-TPE **C** before and **D** after treated with SA (5 × 10^–3^ M) for 24 h

Block copolymer PAGE-Pt-TPE could self-assemble into nanoparticles in aqueous solution, as demonstrated (characterized) TEM with a diameter was about 150 nm (Fig. 2A). The diameter was about 150 nm. Furthermore, the morphology and size changes of PAGE-Pt-TPE nanoparticles after reduction were monitored using TEM and DLS, respectively. PAGE-Pt-TPE nanoparticles exhibited uniform and regular spherical morphology before treated with SA (5 × 10^–3^ M), while as shown in Fig. 2B, it became highly polydisperse in shape and size after treated with SA (5 × 10^–3^ M). Following reduction, the hydrophilic and hydrophobic balance of PAGE-Pt-TPE nanoparticles shifted, leading to a loosening of the nanoparticle structure due to reductive dissociation. Furthermore, the contrast became shallower, consistent with the DLS results (Fig. 2C and D). The values of polydispersity index(PDI) for PAGE-Pt-TPE nanoparticles was 0.69, but after treated with SA, the PDI was 0.82, which was ascribed to disassemble of micelles.

**Fig. 3:**
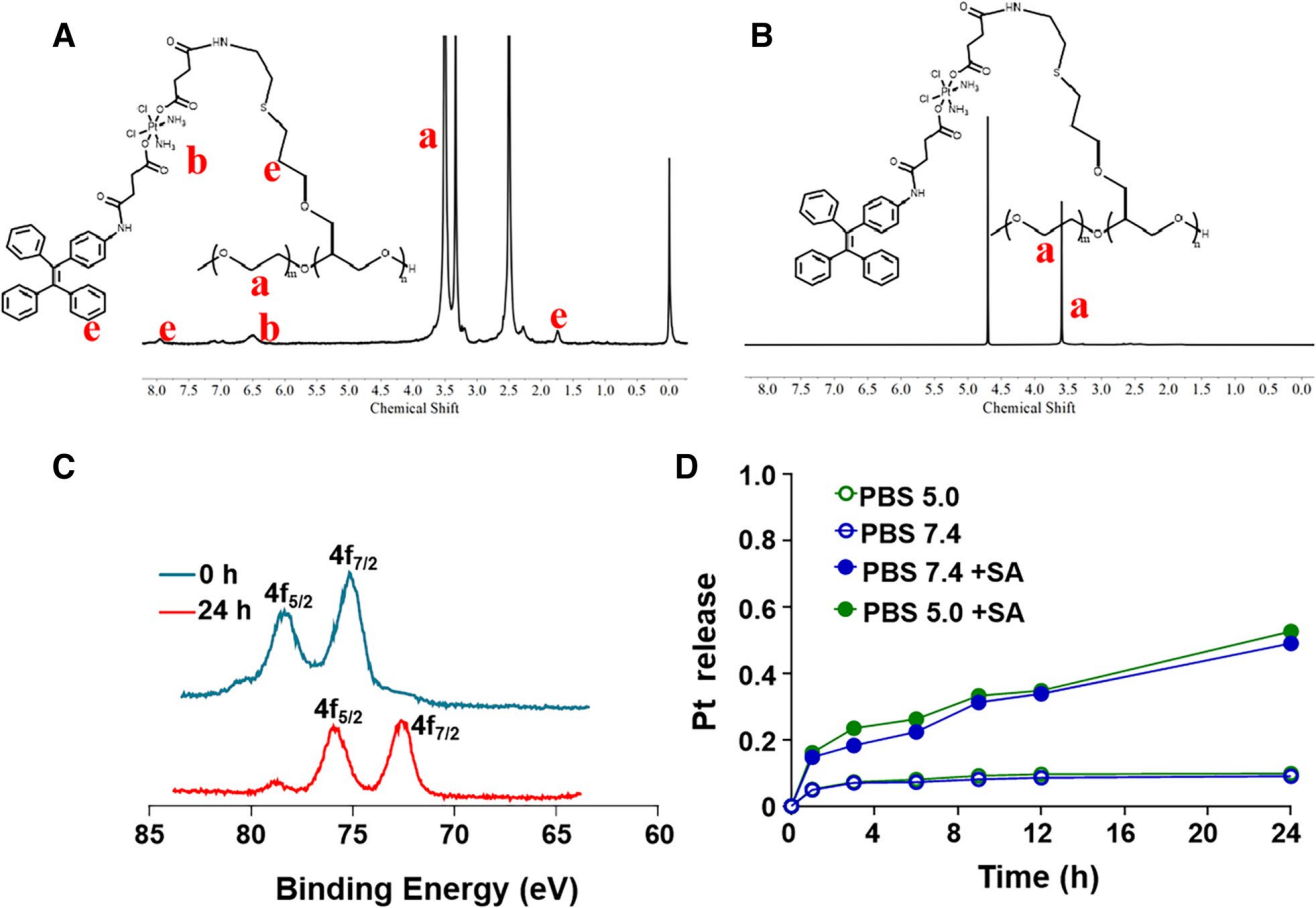
Characterization of PAGE-Pt-TPE and Pt release profiles of PAGE-Pt-TPE. ^1^H NMR spectrum of PAGE-Pt-TPE in **A** DMSO and **B** in D_2_O. **C** XPS curves of Pt_4f_ in PAGE-Pt-TPE micelles before and after treated with SA for 0 h and 24 h; **D** Pt release profiles of PAGE-Pt-TPE in different media (PBS 5.0, PBS 7.4, PBS7.4 with SA and PBS 5.0 with SA)

In order to further prove the successful synthesis of the PAGE-Pt-TPE, it was also confirmed by ^1^H NMR in different solvent. The peak at δ = 7.0 ppm and 8.0 ppm as shown in Fig. 3A, could be assigned to ^1^H of benzene ring in DMSO d_6_. As shown in Fig. S4, the molecular weight of PAGE-Pt-TPE was about 23,411, which indicted that the molecule TPE was conjugated to PAGE-Pt successfully. As previously mentioned, block copolymer PAGE-Pt-TPE could self-assemble into nanoparticles in aqueous solution, which was important for drug delivery system and imaging system. The effective tumor accumulation of nanoparticles/micelles caused by the enhanced permeability and retention (EPR) effect made it possible for passive targeted killing of tumor cells. The successful synthesis of PAGE-Pt-TPE was also proved by ^1^H NMR. As shown in Fig. 3B, Compared with the ^1^H NMR spectrum in DMSO d_6_, all of the peaks of PAGE-Pt-TPE except the peak of the mPEG_5k_ (-OCH_2_CH2-, δ = 3.51 ppm) disappeared in D_2_O, which indicates that PAGE-Pt-TPE self-assembled into micelles in aqueous solution. XPS was also conducted to characterize the change in Pt valence in PAGE-Pt-TPE before and after reduction. As displayed in Fig. 3C, Pt_4f_ in the PAGE-Pt-TPE before reduction exhibited the characteristic binding energies of Pt(IV) (78.5 and 75.2 eV). After reduction, the Pt_4f_ binding energies in the low-molecular-weight species were 75.8 eV and 72.6 eV, which indicates the reduction of Pt(IV) prodrug and the release of the bioactive Pt(II). The release of the bioactive Pt(II) implied fragmentation of the micelles. The reductive Pt release profiles from PAGE-Pt-TPE were further investigated. Though our previous literature research, the intracellular pH value is low. PBS 5.0 solution was used to simulate the release process of PAGE-Pt-TPE in vivo. SA was used to simulate the cell’s reductive environment.^5^ As shown in Fig. 3D, the release percentage of Pt was only 10% after 24 h without reducing agent, while treated with SA for 1 h, approximately 16% of Pt was released from PAGE-Pt-TPE After treated with SA for 24 h, 52% of Pt was released from PAGE-Pt-TPE. These results indicated that PAGE-Pt-TPE was very susceptible to reduction, and the drug release could be precisely controlled by endocytosis.

**Fig. 4:**
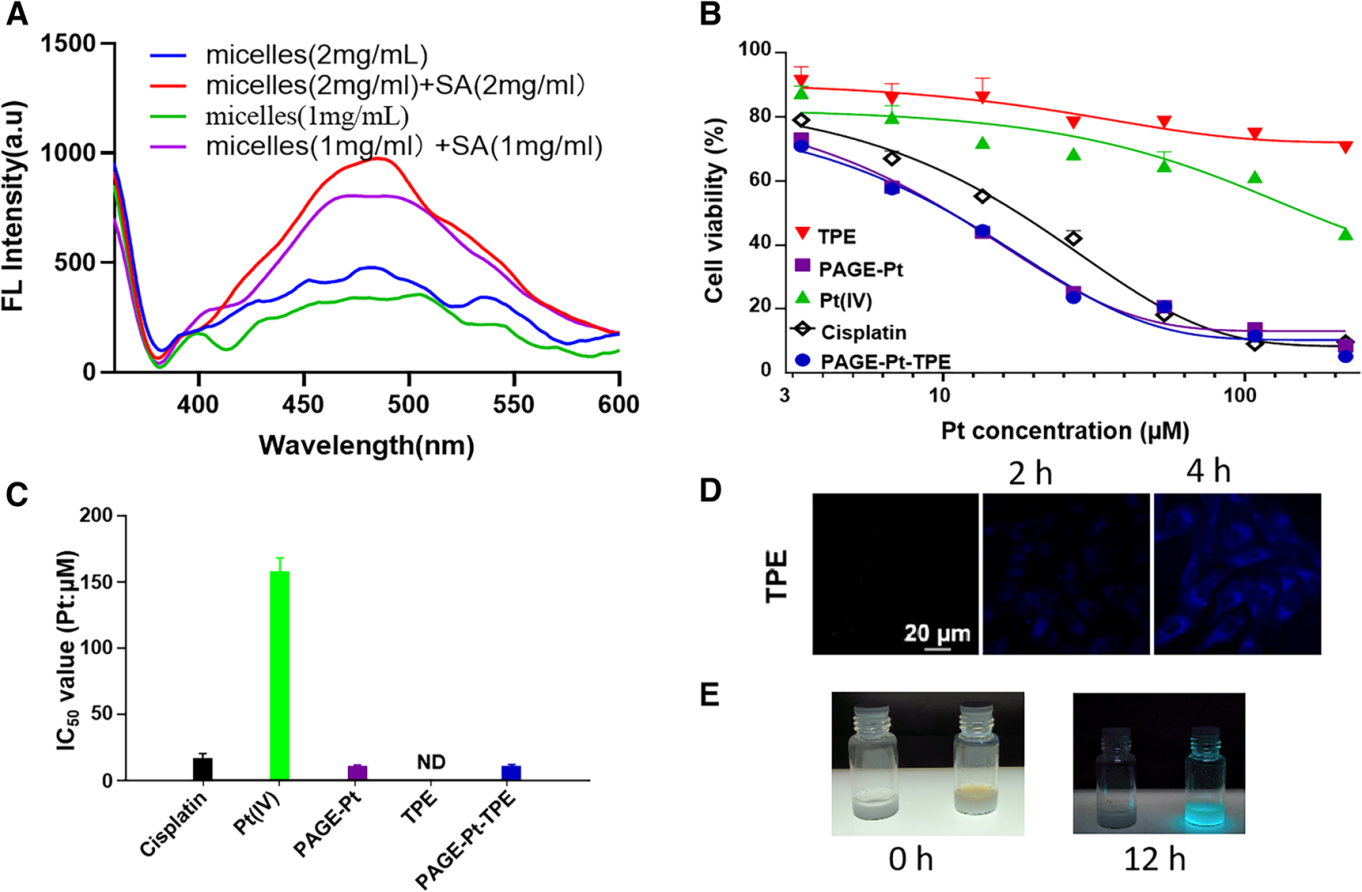
In vitro cytotoxicity profiles and action mechanism of PAGE-Pt-TPE**. A** Fluorescence spectra of PAGE-Pt-TPE before and after treated with different concentration SA; **B** cytotoxicity curves against HeLa cells after 48 h of incubation **C** IC_50_ of different samples **D** CLSM images of HeLa cells pre-treating with PAGE-Pt-TPE incubation for 2 h and 4 h in the dark at 37 ℃. **E** The change of tumor’s supernatant pre-treating with PAGE-Pt-TPE for 12 h

As we know, AIE is a phenomenon that is observed with certain organic luminophores (fluorescent dyes).^7^ Due to ACQ, The photoemission efficiencies of most organic compounds is higher in solution or dispersity than in the solid state. But some organic molecules follows the reverse pattern, being greater in the solid than in dispersity or solution. The effect is attributed to the decreased kinetic energy in the solid. To demonstrate the advantages of combination of TPE and Pt(IV), the luminescence phenomenon of PAGE-Pt-TPE aqueous solutions was detected treated with different concentration SA and the fluorescence spectra of PAGE-Pt-TPE solutions at different concentration. As shown in Fig. 4A, PAGE-Pt-TPE expressed fluorescence intensity, but the fluorescence intensity become stronger when the concentration increase from 1 mg/ml to 2 mg/ml. After treated with SA, PAGE-Pt-TPE expressed intense fluorescence at the same concentration. These results indicate that micelles PAGE-Pt-TPE could improve the solubility of TPE leading to weaker fluorescence, but after treated with reduction, TPE released from PAGE-Pt-TPE and generated agglomeration.

The cytotoxicity tests of PAGE-Pt-TPE against HeLa cells cells were conducted by using cisplatin, Pt(IV), PAGE-Pt, TPE as controls. As shown in Figs. 4B and C, after treatment for 48 h, the IC_50_ value of PAGE-Pt and PAGE-Pt-TPE (10.7 µM) showed much higher cytotoxicity than that of the cisplatin (17.2 µM) and Pt(IV) (158 µM). All the results confirm the cytotoxicity of PAGE-Pt-TPE in vitro. To visualize the internalization process and Imaging effect, the fluorescence monitoring of PAGE-Pt-TPE in cancer cells was investigated by CLSM. After incubation with HeLa cells for 0 h, none blue fluorescence from TPE was observed in cytoplasm (Fig. 4D). But after incubation for 2 and 4 h, strong blue fluorescence was observed in cytoplasm. TPE reflects the process of drug release and the fluorescence intensity can also reflect the sensitivity of tumor cells to drugs.

After volume of subcutaneous U14 tumor model reach to 500 mm^3^, the tumor was collected from sacrificed mice. The supernatant was treated with PAGE-Pt-TPE for 12 h at 37 ℃ in a shaking culture incubator. As shown in Fig. 4E, both supernatant without any treatment and treated with PAGE-Pt-TPE nanoparticles for 0 h had no fluorescence under light irradiation at 365 nm. But 12 h later, the sample treated with PAGE-Pt-TPE emitted strong fluorescence, while the other sample did not. Therefore, PAGE-Pt-TPE micelles can release TPE at tumor microenvironment. At the same time, drug release and distribution were tracked.

## Conclusion

In summary, we have developed a PAGE-Pt-TPE micelle system for precise chemotherapy guided by AIE imaging without ACQ. The PAGE-Pt-TPE system enables the reduction-triggered release of the Pt(IV) prodrug, resulting in the release of bioactive Pt(II) drug within cancer cells or at the tumor site. Additionally, the cleaved polymer segment further reduces to produce TPE, leading to AIE both in vitro and in vivo. Moreover, these micelles hold potential for diagnostic applications. For instance, if modified with targeted molecules or specific antibodies, they can accumulate at specific sites and emit a fluorescent signal. It will spread rang of application if broaden the wavelength range of TPE.

## Author*’* contributions

ZGW wrote the main manuscript text; GLL, QHZ, GYF and ZLY prepared all the figures. All authors reviewed the manuscript.

### Supplementary Information

**Supplementary Material 1. **

## Data Availability

The datasets used and/or analysed during the current study available from the corresponding author Guojun Zhang (guojun.zhang@ccmu.edu.cn) on reasonable request.
