# Supplementary material for: Reductive prodrug and AIE copolymer nanoparticle for monitoring and chemotherapy
Source: BMC Cancer. 2024 Mar 26;24:382. doi: 10.1186/s12885-024-12135-7 (PMC10964534; doi:10.1186/s12885-024-12135-7)
Supplement: Supplementary file 1 — Supplementary Material 1. [file 12885_2024_12135_MOESM1_ESM.doc]

**Reductive Prodrug and AIE Copolymer Nanoparticle for** **Monitoring and Chemotherapy**

Zigui Wang ^1,2,3,4^ Guilin Li ^4^ Qiaohui Zhao^4^ Guangyu Fu^5^ Zengli Yang^5^ Guojun Zhang ^1,2,3 *^

1. Department of Clinical Diagnosis, Laboratory of Beijing Tiantan Hospital, Capital Medical University, Beijing, China;

2. NMPA Key Laboratory for Quality Control of In Vitro Diagnostics, Beijing, China;

3. Beijing Engineering Research Center of Immunological Reagents Clinical Research, Beijing, China;

4. Zhengzhou Immunobiotech Co.,Ltd, Zhengzhou 450016, PR China.

5. Autobio Diagnostics Co. , Ltd, Zhengzhou 450016, Henan, China.

*Corresponding authors at: Department of Clinical Diagnosis, Laboratory of Beijing Tiantan Hospital, Capital Medical University, Beijing, China (Guojun Zhang).

1. mail addresses: guojun.zhang@ccmu.edu.cn (Guojun Zhang).


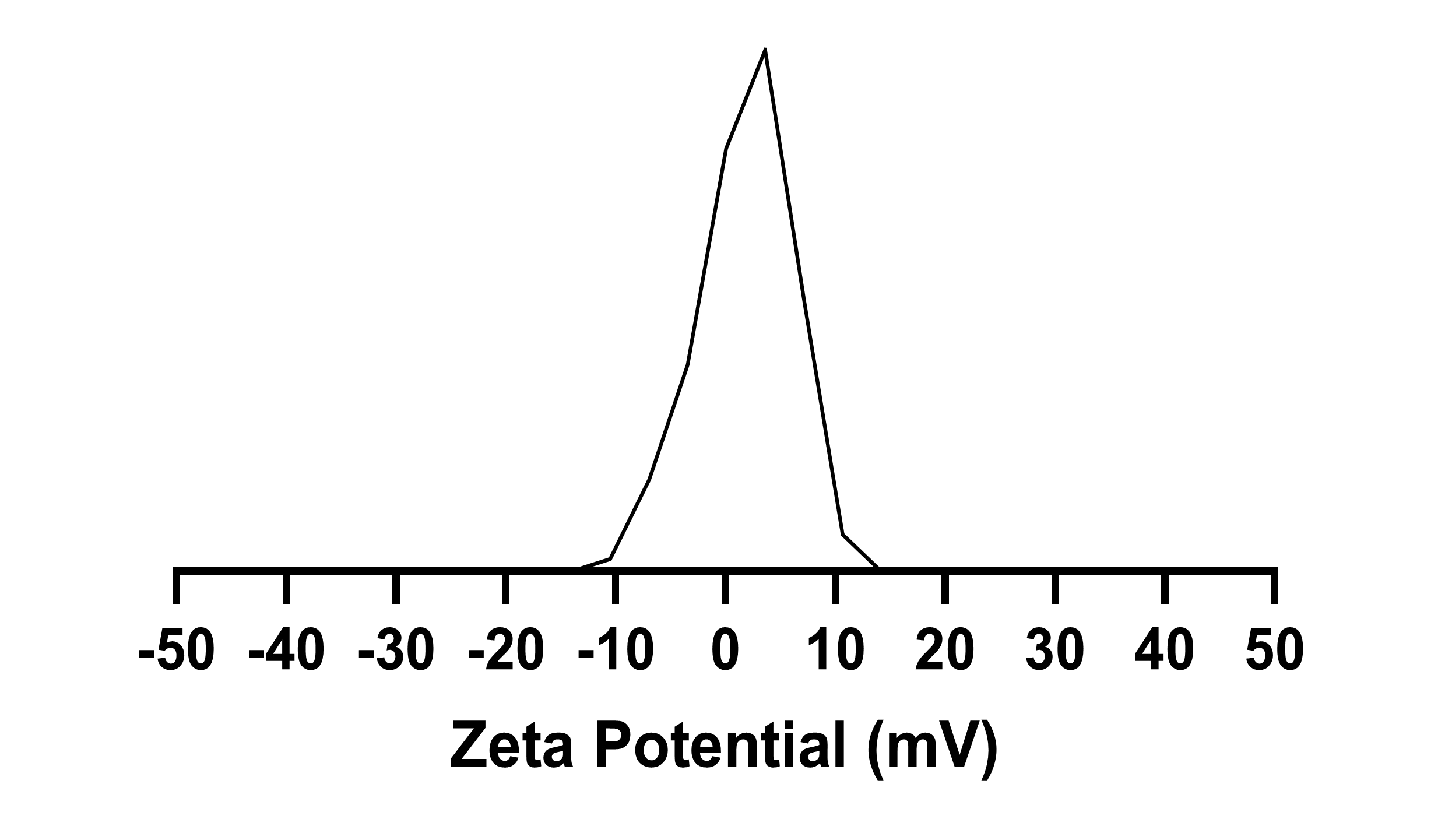


**Fig.S1** the Zeta potential of PAGE-Pt-TPE nanoparticles.


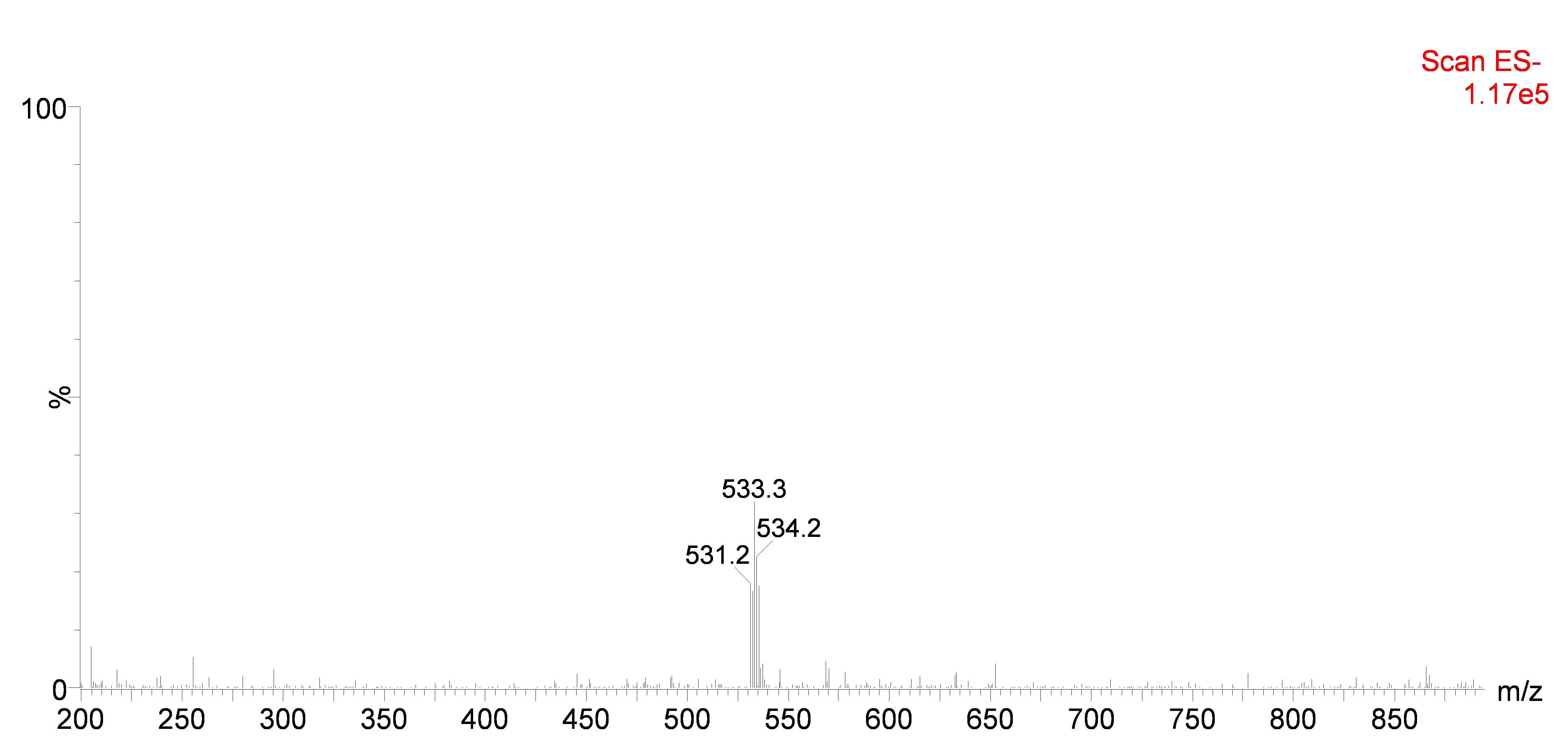


Fig .S2 The ESI-MS spectral of Pt(IV)-COOH


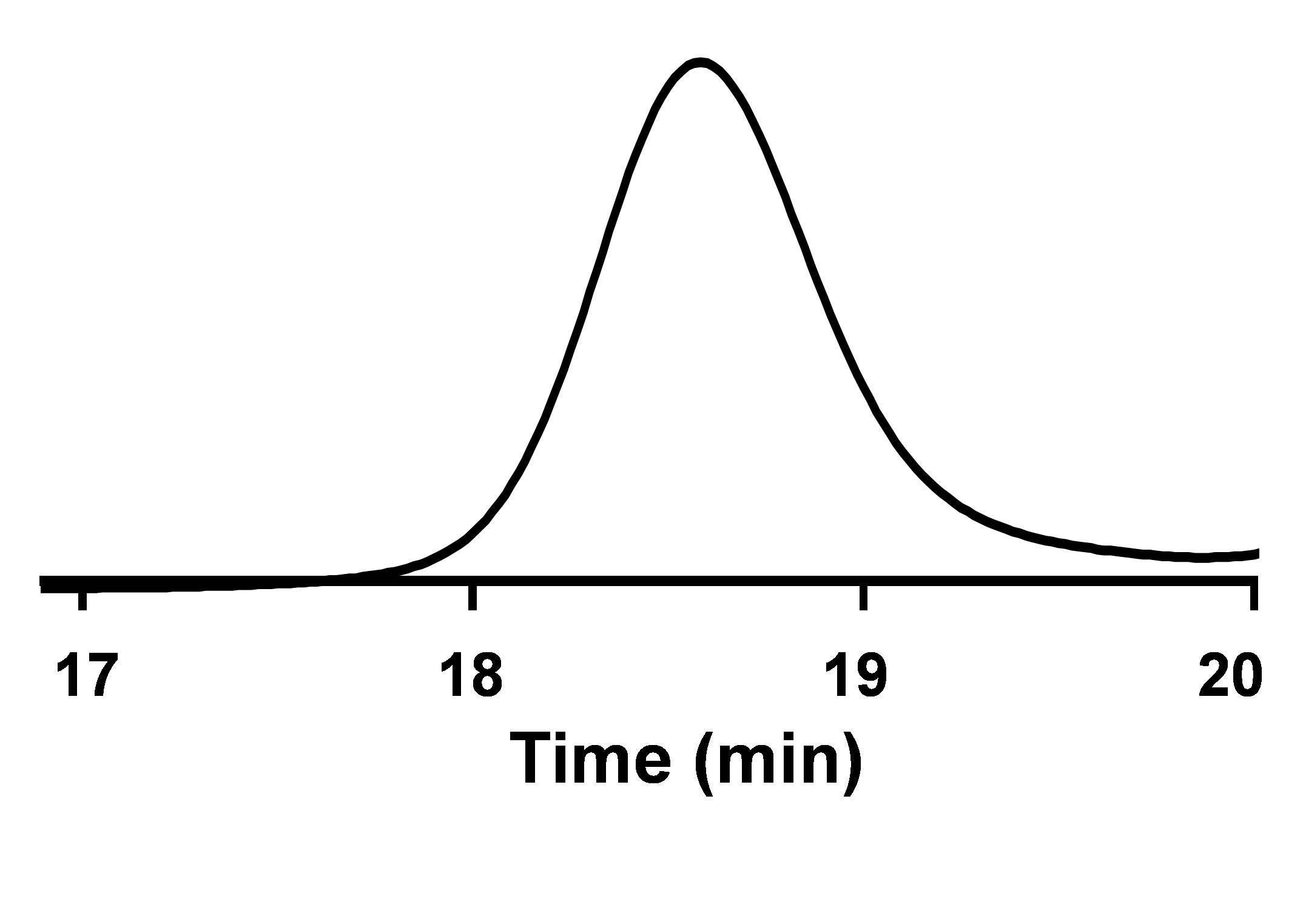


**Fig.S3** The molecular weight of PAGE-Pt(Mn=14500 Da, PDI=1.13).


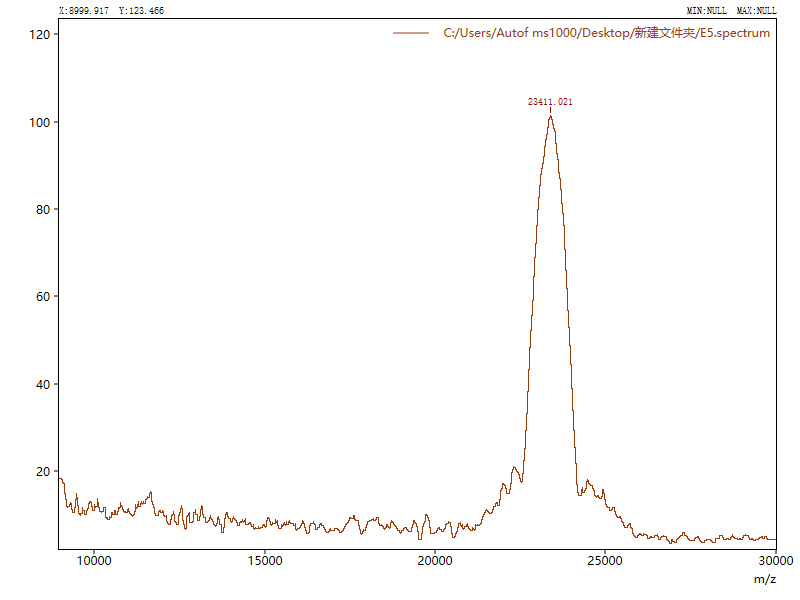


**Fig.S4** The molecular weight of PAGE-Pt-TPE was conducted by MALDI-TOF-MS.
